# Cherokee County Medical Society

**Published:** 1891-07

**Authors:** 


THE CHEROKEE COUNTY MEDICAL SOCIETY is one of the foremost, most progressive and enlightened organizations that have sprung up "under the influence of spirited journalism" in Texas. They are hard workers and are making a record.

At a recent meeting held at Alto, Cherokee county, Dr. J. H. McCord in the chair,—a full report of which was sent the JOURNAL by the courteous Secretary, Dr. J. K. Frazier,—there was an unusually large attendance and an animated discussion. The subject was, "Is small-pox contagious during the initiatory fever, or vesicular stage?" A great diversity of opinion was brought out, and as some of the views presented were original and unique the JOURNAL much regrets its inability to publish the report in full. It will be remembered that Dr. B. F. Brittain, a member of this society, read a paper on the subject at the Waco meeting of the State Medical Association which created some interest. The Cherokee Society had a committee on "Collective Investigation" to investigate the subject, and it was discussed exhaustively.

Dr. Frazier held, and we agree with him, that small-pox is contagious "from the initiatory fever, on through all the stages, until the patient is well." This opinion was shared by Drs. Osborn, Roland, Quinn, Ramsay, Kirksey, Burton and others, but many of the members differed with him, and Dr. J. M. Brittain held that the disease is not contagious during the first stage; his experience justified such belief. Dr. B. F. Brittain—who took that position in his Waco paper—says it is not contagious during the initiatory fever or up to the close of the vesicular stage, and cited cases of exposure which bore him out in his position.

But Dr. Guinn, of Alto, took a medium and safe ground, and is reported by the Secretary to have held that "it is, and is not contagious."

The JOURNAL ventures to suggest that there *is* a time when the disease is not contagious, or when the danger is reduced to a minimum,—not alluded to in the discussion; that is, when all exposed to the disease in any of its stages are *thoroughly vaccinated!* In view of the almost unaminous testimony to the efficacy of vaccination and the undoubted contagiousness of the disease, it is remarkable that any one should neglect the precaution of thorough vaccination even a day.

At the meeting several interesting papers were read and discussed, and our correspondent reports a gratifying degree of interest—almost enthusiasm—in the good work. Let other societies emulate the example of Cherokee county.

